# Cognitive and affective trait and state factors influencing the long-term symptom course in remitted depressed patients

**DOI:** 10.1371/journal.pone.0178759

**Published:** 2017-06-02

**Authors:** Christina Timm, Bettina Ubl, Vera Zamoscik, Ulrich Ebner-Priemer, Iris Reinhard, Silke Huffziger, Peter Kirsch, Christine Kuehner

**Affiliations:** 1 Research Group Longitudinal and Intervention Research, Department of Psychiatry, Central Institute of Mental Health, Medical Faculty Mannheim, Heidelberg University, Mannheim, Germany; 2 Department of Clinical Psychology, Central Institute of Mental Health, Medical Faculty Mannheim, Heidelberg University, Mannheim, Germany; 3 Karlsruhe Institute of Technology, Institut für Sport und Sportwissenschaften, University of Karlsruhe, Karlsruhe, Germany; 4 Department of Psychosomatic Medicine and Psychotherapy, Central Institute of Mental Health, Medical Faculty Mannheim, Heidelberg University, Mannheim, Germany; 5 Department of Biostatistics, Central Institute of Mental Health, Medical Faculty Mannheim, Heidelberg University, Mannheim, Germany; Erasmus University Rotterdam, NETHERLANDS

## Abstract

**Background:**

Major depressive disorder (MDD) is characterized by a high risk for relapses and chronic developments. Clinical characteristics such as residual symptoms have been shown to negatively affect the long-term course of MDD. However, it is unclear so far how trait repetitive negative thinking (RNT) as well as cognitive and affective momentary states, the latter experienced during daily-life, affect the long-term course of MDD.

**Method:**

We followed up 57 remitted depressed (rMDD) individuals six (T2) and 36 (T3) months after baseline. Clinical outcomes were time to relapse, time spent with significant symptoms as a marker of chronicity, and levels of depressive symptoms at T2 and T3. Predictors assessed at baseline included residual symptoms and trait RNT. Furthermore, momentary daily life affect and momentary rumination, and their variation over the day were assessed at baseline using ambulatory assessment (AA).

**Results:**

In multiple models, residual symptoms and instability of daily-life affect at baseline independently predicted a faster time to relapse, while chronicity was significantly predicted by trait RNT. Multilevel models revealed that depressive symptom levels during follow-up were predicted by baseline residual symptom levels and by instability of daily-life rumination. Both instability features were linked to a higher number of anamnestic MDD episodes.

**Conclusions:**

Our findings indicate that trait RNT, but also affective and cognitive processes during daily life impact the longer-term course of MDD. Future longitudinal research on the role of respective AA-phenotypes as potential transdiagnostic course-modifiers is warranted.

## Introduction

Major depressive disorder (MDD) is a highly prevalent mental disorder characterized by a high risk of chronicity and relapse [1–3]. In light of the unfavorable long-term course of MDD, it is important to identify risk factors that contribute to the clinical course of the disorder. In previous studies, clinical characteristics such as the number of previous episodes and subclinical residual symptoms have been identified as relevant course modifiers [2, 4, 5]. Less is known about the influence of maladaptive habitual cognitive thinking on the long-term course of depression. First evidence suggests that rumination, a cognitive trait defined as a negative pattern of responding to distress by repetitively and passively focusing on the meanings, causes, and consequences of one’s depressive symptoms [6, 7] predicts symptom severity [8, 9], as well as a chronic symptom course in depression [10]. Maladaptive habitual thinking styles like rumination or worry are subordinate constructs of the broader concept of trait repetitive negative thinking (RNT) [11, 12]. RNT is defined as repeatedly occurring, uncontrollable, negative and abstract thoughts from which it is difficult to disengage and has been considered to represent a potential transdiagnostic cognitive risk factor for various mental disorders [12]. Studies suggest that trait RNT is cross-sectionally associated with symptoms and treatment outcomes of depression and anxiety [11, 13, 14]. Only one study so far has identified the predictive validity of RNT for the 3-year course of depressive symptoms in a nonclinical sample [15]. Comparable data in clinically depressed samples is still missing.

In contrast to macro-level risk factors such as trait RNT, possible dysfunctional cognitive and affective momentary state variables reflect mental states at the micro-level of moment-to-moment experiencing during daily life (cf. [16, 17, 18]). The investigation of such momentary experiences and their variability over the day appears important, because there is increasing evidence that they may affect the course of psychopathology at the macro-level (for a review see [18]). A promising approach to examine micro-level risk factors in depression is ambulatory assessment (AA) [16, 19]. Here, individual data is repeatedly collected over a given period of time and in different contexts. During AA, individuals rate their affective states and current thoughts or activities. Thereby, it is possible to assess state-like vulnerability factors in a naturalistic context and to investigate their predictive value for the future course of illness [18, 20].

Several studies have investigated affective state variables showing that MDD patients report less positive and more negative affect in daily life compared to healthy controls [21, 22]. Recent efforts undertaken to test predictive effects of momentary affective processes on the course of depression yielded compelling results, substantiating that individual responses to stressful and rewarding events during daily life are associated with future depressive symptoms [18, 23, 24]. In addition, depressive individuals seem to display a distinct affective dynamic over the day [25–27]. Mood instability has predominantly been studied in patients with other psychopathology, such as those with borderline personality disorder [28, 29], but first evidence suggests that, for example, variations in negative affect may also have detrimental effects on the clinical course [30] and treatment response [31] in MDD. Furthermore, by applying a dynamic system framework, we could previously show that high entropy, i.e. frequent and unpredictable changes in the interplay between momentary mood and rumination, was related to an increase of depressive symptoms during six months in remitted depressed (rMDD) individuals [32].

There is a clear lack of AA-studies investigating momentary cognitive processes as a potential course-modifying vulnerability factor. One such possibly important cognitive momentary risk factor is daily-life rumination. Up to now, momentary rumination has been predominantly examined in non-clinical samples, thereby demonstrating an increase in depressive symptoms, e.g. sleep disturbances [33] or negative affect [34] over a short period. Furthermore, depressive symptoms in a student sample were predicted by the level of momentary rumination [35]. Compared to control participants, clinically depressed and anxious individuals are characterized by higher levels of momentary rumination [36]. In a previous study [37], we found that momentary rumination was associated with higher cortisol secretion over the day in rMDD individuals, demonstrating prolonged activation of the bodily stress system in response to rumination with possible detrimental effects on the further course of depression. However, studies explicitly investigating the impact of momentary rumination and its instability over the day on the long-term course of depression in clinical samples are lacking.

In summary, it appears of high importance to investigate the possible predictive value of both trait RNT and momentary affective and cognitive states during daily life for the long-term course of depression. Therefore, the present study aimed to test the predictive validity of such traits and states at baseline on course-related outcomes, i.e., time to relapse, chronicity, and levels of depressive symptoms over three years, in individuals with rMDD. Although clinical predictors were not the focus of the study, we chose to additionally include residual symptom levels at baseline, which are considered a potent clinical predictor for relapse and other poor outcomes in depression [38–40], and which also overlap with other clinical predictors such as the severity of the previous index episode [41].

Based on previous research showing that both high entropy and variability in daily-life affect appears to be linked to an increase in depressive symptoms in rMDD individuals [30, 32] we expected that a fluctuating course (i.e., relapses) would be predominantly predicted by instability measures of daily life affect and cognition. Up to now, these variability measures have not been tested for the more stringent criteria of relapse. In contrast, first evidence suggests a link between habitual rumination and a chronic symptom course [10] leading to the hypothesis that chronicity and symptom level outcomes would predominantly be predicted by trait RNT.

## Methods

### Procedure

Participants were recruited by announcements in local newspapers and on the homepage of the Central Institute of Mental Health (CIMH), Mannheim, Germany. After a telephone prescreening, preliminary eligible participants were invited to the CIMH, and a trained clinical psychologist administered the Structured Clinical Interview for DSM-IV axis I (SCID-I, [42], see below) as part of the baseline interview (T1) to assess in- and exclusion criteria. All rMDD individuals had to fulfill either the criteria for at least two lifetime MDD episodes or a previous chronic MDD of at least two years duration. At T1, they had to be remitted from the last episode, i.e., did not fulfill the criteria of a Major Depressive Episode according to DSM-IV, for at least two months. Exclusion criteria were non-affective psychotic disorders, bipolar disorder, substance dependence, current substance abuse, generalized anxiety disorder, and current obsessive-compulsive, posttraumatic stress, and eating disorder according to DSM-IV. In addition to rMDD participants, the study also recruited healthy individuals, which are, howerver, not subject to the present analyses.

Demographic, clinical, and cognitive trait predictors in rMDD individuals were assessed at T1, followed by ambulatory assessment (AA) of affective and cognitive state factores, assessed during the days immediately following the diagnostic baseline interview (see paragraph “Predictor variables”). Follow-ups on the course of clinical depression and depressive symptoms took place at six (T2) and 36 months (T3) after baseline and were conducted by a trained clinical psychologist during a telephone interview. At all measurement points, we assessed diagnostic status and symptom levels and, at T2 and T3, the course of depression since the last assessment.

Importantly, the present study sample of rMDD participants consisted of two consecutively recruited subsamples (subsample 1: Oct 2010 to Apr 2011, subsample 2: Nov 2011 to Nov 2012) from an overarching study. The two subsamples underwent different functional magnetic resonance imaging (fMRI) experiments, conducted after the ambulatory assessment days, which are not subject to the present analyses (see, [43]). In addition, the AA of subsample 1 was restricted to the assessment of naturally occurring mood and rumination over the day, whereas in subsample 2 an additional rumination versus mindful self-focus manipulation during AA was conducted. The two AA-procedures are described in the supporting information (S1), together with detailed analyses regarding the comparability of subsample 1 and 2 and the legitimation for their combined inclusion for the present long-term analyses. The study was in accordance with the Declaration of Helsinki and was approved by the local Ethics Committee of the University of Heidelberg. All participants gave written informed consent.

### Sample recruitment and attrition

Originally, 101 individuals were contacted during the telephone prescreening, of whom eight participants were excluded (n = 2 fulfilling fMRI exclusion criteria (see above), n = 6 declined to participate). At the diagnostic baseline session (T1), 27 individuals were excluded due to diagnostic exclusion criteria, resulting in a sample of n = 66 remitted depressed (rMDD) individuals with whom the comprehensive baseline assessment was carried out. Of those, one participant dropped out after T1 (could not be reached), and eight individuals dropped out after T2 (n = 1 moved abroad, n = 7 could not be reached). Consequently, 57 of initially recruited 66 participants (86.4%) provided data for all measurement points and were included in the present analyses. Demographic and clinical characteristics at study entry did not differ significantly between participants participating in both follow-ups and those who dropped out during the study, with one exception: there was a significant dropout of participants with lower education levels (n = 9 drop outs vs n = 57 completers: school education < 10y: χ^2^ = 5.33, *p* = .021). In contrast, individuals who completed the study and those who dropped out did not differ with regard to clinical variables at T1 (n = 9 drop outs vs n = 57 completers: BDI-II: t = -.225, *p* = .823, MADRS: t = -.431, *p* = .668, number of previous episodes: χ^2^ = .085, *p* = .771).

### Outcome measures

The following three outcome measures related to the course of illness were investigated: time to relapse, chronicity and depressive symptom levels. The current diagnostic status at all measurement points (T1-T3) was determined with the SCID-I [42]. If an individual met criteria for a current major depressive episode (MDE) during the follow-up assessments (T2, T3), we coded the number of weeks since the beginning of the current episode. Additionally, we coded the number of weeks related to the beginning and ending of those depressive episodes occurring exclusively during the follow-up intervals. Thereby, we assessed both pure interval episodes during the T1-T2 and T2-T3 intervals and episodes that were present at T2 and T3.

#### Time to relapse

For the outcome “time to relapse”, we calculated the number of weeks to the first MDE after T1. Relapse was defined as the recurrence of a depressive syndrome fulfilling criteria of a MDD after T1. This term was used as an umbrella term for relapses (i.e. within six months after remission, n = 1) and recurrences (i.e. after six months, n = 20). Due to the limited sample size, we combined these two outcomes to increase statistical power.

#### Chronicity

Chronicity was defined as the percentage of weeks spent with significant symptoms (i.e., without distinct symptom relieve) after T1 using the information of the expanded SCID-I at T2 and T3.

#### Depressive symptom levels

As a measure of levels and course of depressive symptoms we used the self-rated Beck Depression Inventory Revised (BDI-II, [44]) and the interviewer-rated Montgomery and Asberg Depression Rating Scale (MADRS, [45]), which both have shown good reliability, validity and sensitivity to symptom changes [46–48]. For statistical analysis, we calculated an overall composite score for depressive symptoms by averaging the z-standardized BDI-II and MADRS scores both at T2 and T3, as done in previous research (e.g., [37, 49]).

### Predictor variables

#### Demographic and clinical variables

Demographic variables included age, gender and education status. Clinical variables included residual depressive symptoms at baseline (T1) and current use of medication at T1 (the latter to check for possible confounding effects with residual symptoms and outcome measures). Depressive symptoms at T1 were calculated as a composite score of the MADRS and the BDI-II scores (see above).

#### Trait repetitive negative thinking

Trait repetitive negative thinking (RNT) was measured with the Perseverative Thinking Questionnaire (PTQ, [12, 50]), which is conceptualized as a content-independent measure of RNT. Individuals are asked how they *typically* think about negative experiences or problems. Research has shown that the PTQ has good psychometric properties, i.e. internal consistency, stability, factor-structure and construct validity [50, 51].

#### Affective and cognitive state variables

After the baseline interview, affective and cognitive state variables were assessed by AA during the following days. In subsample 1 (n = 28), participants were assessed with personal digital assistants (PDAs, Palm Tungsten E2, Palm Inc.) and the software IzyBuilder (IzyData Ltd., Fribourg, CH) over two consecutive weekdays with ten assessments per day (for detailed description see [37]). AA in subsample 2 (n = 29), was conducted over four consecutive weekdays with ten assessments per day using smartphones (HTC Touch Diamond 2) and the software MyExperienceIDE by movisens GmbH (Karlsruhe, Germany). The two subsamples completed identical affective and cognitive AA measures (for further details, see supporting information S1).

Momentary mood was measured with six bipolar items specifically developed for AA [52] that were collapsed into the three scales “valence” (items “content-discontent”, “unwell-well”), “calmness” (items “agitated-calm”, “relaxed-tense”) and “energetic arousal” (items “tired-awake”, “full of energy-without energy”). Three items (one per subscale) were recoded so that higher values indicate a more positive mood component. These subjective mood scales have shown good reliability and validity [52].

**Fig 1:**
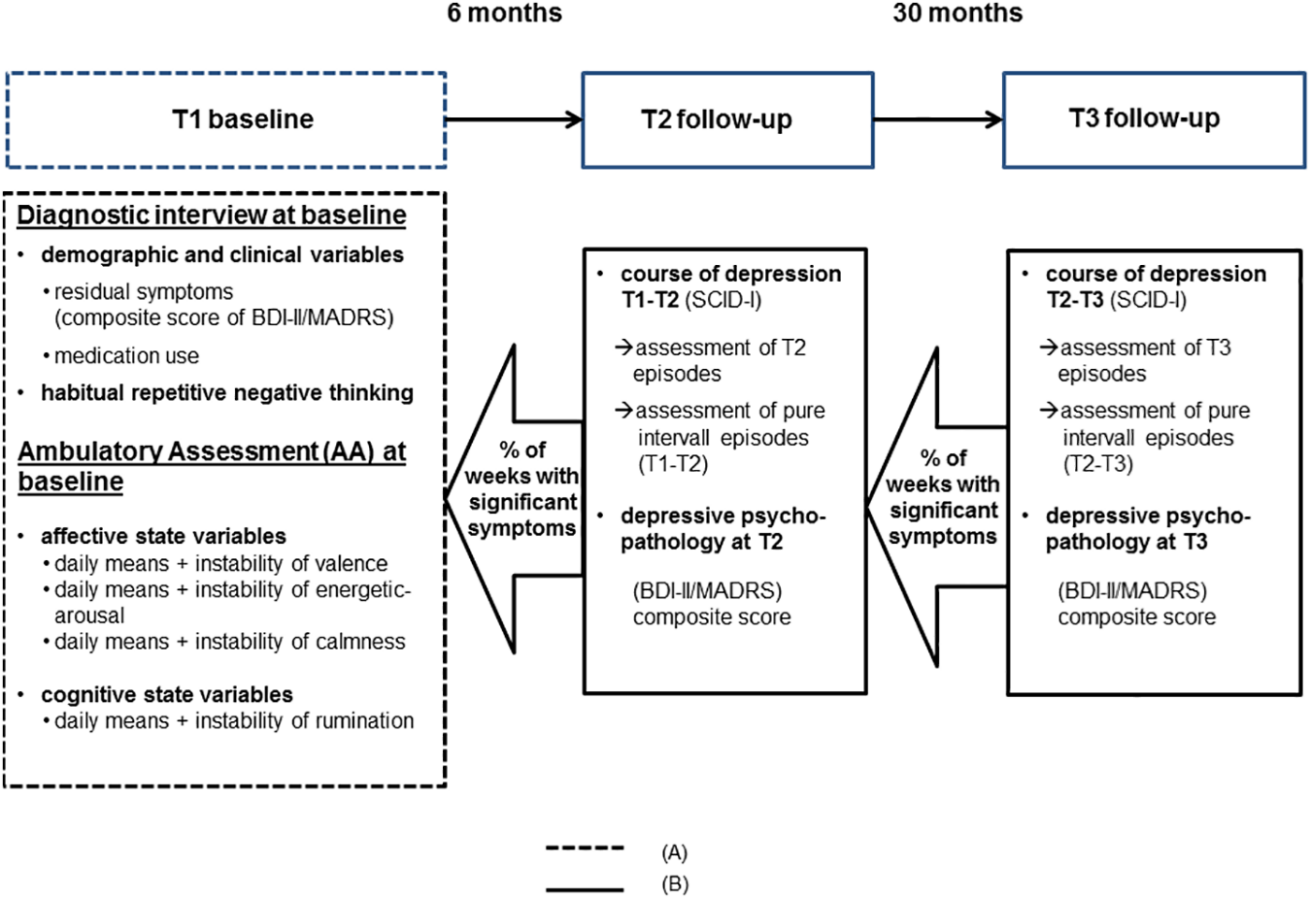
Study design. Baseline predictors. (B) Diagnostic information to define outcome variables. BDI-II BeckDepressionInventory II. MADRS Montgomery and Asberg Depression Rating Scale. SCID-I Structured Clinical Interview for DSM-IV Axis 1.

Momentary ruminative self-focus was operationalized with the average score of two items developed by Moberly and Watkins [34]: “At the moment, I am thinking about my problems” and “At the moment, I am thinking about my feelings”. These items proved to be suitable for studies with AA-designs [34, 53]. Fig 1. shows the overall study design.

### Statistical analyses

For the AA predictors, we used aggregated scores, i.e. overall means over the respective assessment days, of the momentary scales (valence, calmness, energetic arousal, rumination) together with their averaged instability scores. The latter were calculated according to von Neumann and collegues [54] using the *mean squared successive difference* (MSSD, see Eq (1)).

$$MSSD(X)=\frac{\sum_{i=2}^{n}{\left(X_{i}-X_{i-1}\right)}^{2}}{n-1}$$ (1)

The MSSD represents instabilities in a time series as an average of all squared successive changes over time. Thereby, MSSD takes into account three components of instability: temporal order, amplitude and frequency of change. A high MSSD score reflects high temporal instability as a result of high amplitudes and high frequency of, e.g., mood swings over the day, corresponding to low temporal dependency [55–57]. MSSD is highly correlated with the standard deviation (SD) [55]. However, compared to variability, defined as the dispersion of scores from a central tendency, MSSD takes into account gradual shifts in means over time [55]. For example, while the variability of the mood component “energetic arousal” [52] would be similar for a person with two extreme states of “energetic arousal” in the morning and evening compared to a person with frequent, less extreme swings of “energetic arousal” over the day, MSSD reflects these differences [55]. MSSDs were calculated for each momentary mood component and for momentary rumination.

For each outcome, we present the results of simple regression analyses, i.e., the association between outcome and each predictor separately in a first step. To determine the independent contribution of each predictor, we then entered those predictors with a *p*-value of < .05 simultaneously into a multiple model using backward elimination of predictors, and retained all predictors in the model that significantly (*p* < .05) contributed to the respective outcome. Effects of predictors on time to relapse were estimated using Cox regressions. To predict chronicity, we applied linear regression analyses with the percentage of weeks spent with significant symptoms as the dependent variable. This outcome was positively skewed and therefore log-transformed to yield a better approximation of a normal distribution. Effects on depressive symptom levels were analyzed by hierarchical linear models with depressive composite scores at T2 and T3 as outcome. For the latter analyses, we first tested main effects of individual predictors and their interaction effect with time (T2, T3) in separate regression models. If the interaction term did not reach statistical significance, we removed this term from the respective models. In a next step we included all significant predictors and significant interaction effects in a multiple model.

In all multiple models described above, subsample status was included as a covariate to control for possible confounding effects of this variable (for further information, see supporting information S1). All statistical analyses were performed using the statistical software IBM SPSS Version 20.

## Results

### Participants’ characteristics

Demographic, clinical, as well as cognitive trait and state characteristics are presented in Table 1. The mean age at MDD-onset in rMDD participants was 23.8 years (SD = 11.2), and 70% of these individuals reported at least 3 lifetime MDD episodes upon entering the study.

**Table 1:** Demographic, clinical, cognitive trait and affective and cognitive state characteristics in remitted depressed patients (rMDD) at baseline.

	rMDD (n = 57)	
	Mean (SD) / %	Range (min–max)
**demographic**		
age (SD)	43 (10.3)	(23–55)
gender (female %)	70.2%	
education (% > 10 years)	63.2%	
work situation (% in regular job or education)	68.4%	
marital status (% married or living together)	49.1%	
**clinical**		
BDI-II^1^	10.6 (8.8)	(0–36)
MADRS^2^	5.8 (4.8)	(0–22)
current psychotherapy	22.8%	
current psychotropic medication^3^	24.6%	
number of previous episodes	3.8 (1.9)	(1–7)
duration of previous hospitalization (weeks)	9.3 (12.9)	(0–75)
residual symptom levels (composite score BDI-II^1^,MADRS^2^)	0.5 (1.0)	(-0.8–4.1)
**trait repetitive negative thinking**		
PTQ^4^	30.8 (14.5)	(0–60)
**affective state variables (AA)**^5^^,^^6^		
daily-life valence	3.7 (0.9)	(2.4–5.9)
daily-life calmness	3.4 (0.9)	(1.6–5.7)
daily-life energetic arousal	3.4 (0.8)	(1.7–5.4)
instability of daily-life valence	1.3 (0.9)	(0.04–3.8)
instability of daily-life calmness	1.6 (1.2)	(0.2–6.6)
instability of daily-life energetic arousal	1.6 (1.1)	(0.2–5.0)
**cognitive state variables (AA)**^5^^,^^6^		
daily-life rumination	1.6 (1.1)	(0–4.2)
instability of state rumination	2.9 (2.6)	(0–9.2)

^1^ BDI-II BeckDepressionInventory II

^2^ MADRS Montgomery and Asberg Depression Rating Scale

^3^ selective serotonin reuptake inhibitors (SSRIs): n = 4, serotonin-norepinephrine reuptake inhibitors (SNRIs): n = 5, noradrenergic and specific serotonergic antidepressant: n = 2, selective serotonin and noradrenalin reuptake inhibitor (SSNRI): n = 1, tricyclic antidepressants (TCAs): n = 1, amber: n = 1, Lithium: n = 2, atypical antipsychotic medication: n = 1; anticonvulsant: n = 1; n = 4 participants with multiple prescriptions

^4^ PTQ Perseverative Thinking Questionnaire

^5^AA Ambulatory Assessment

^6^averaged across assessment days.

During the 3-year follow-up period, 28 (49.1%) of the originally remitted depressed patients had suffered at least one relapse into a major depressive episode. Furthermore, the mean proportion of weeks spent with significant symptoms during the 3-year interval was 34.9% (range 1%–95%) with 28% spending more than 50% of the weeks with significant symptoms.

### Predictors of time to relapse

Simple Cox regression analyses revealed that higher levels of residual depressive symptoms, as well as higher instability of momentary rumination (AA) and of valence (AA) predicted a shorter time to relapse (Table 2). We further conducted correlational analyses on the relationship between number of previous episodes and predictors. The respective coefficients are: r = 0.246 (*p* = .067) for residual symptoms; r = 0.308 (*p* = .022) for instability of momentary valence and r = 0.468 (*p* < .001) for instability of momentary rumination.

**Table 2:** Simple and multiple regression models for time to relapse, chronicity, and depression scores (T2, T3) in remitted depressed patients (rMDD).

outcome variables	shorter time to relapse after T1^a^ (n = 57)		chronicity/ % of weeks with significant symptoms after T1^b^ (n = 57)		composite depression score (BDI-II/MADRS) T2, T3^c^ (n = 57)	
predictors	simple	multiple^f^	simple	multiple^f^	simple	multiple^f^
	B (SE)	B (SE)	B (SE)	B (SE)	B (SE)	B (SE)
**demographics**						
Age	0.003 (0.019)	-	- 0.001 (0.014)	-	0.007 (0.010)	-
sex (1 = m, 2 = f)	-0.518 (0.388)	-	- 0.547 (0.313)	-	0.236 (0.239)	-
education level (0 = ≥10y, 1 = <10 y)	0.238 (0.040)	-	0.074 (0.304)	-	-0.034 (0.229)	-
**clinical variables**						
medication use (0 = no, 1 = yes)	-0.116 (0.437)	-	-0.388 (0.337)	-	-0.366 (0.303)	-
residual symptoms at baseline	0.498 (0.173)**	0.585 (0.187)**	0.360(0.135)**	-	0.522(0.082)***	0.502(0.077)***
**trait repetetive negative thinking**	0.020 (0.014)	-	0.037(0.039)***	0.032(0.009)***	0.024(0.007)***	-
**affective state variables (AA)**^d^						
valence	-0.289 (0.240)	-	-0.293 (0.160)	-	-0.386(0.113)**	-
energetic arousal	-0.189 (0.248)	-	-0.366(0.181)*	-	-0.436(0.128)**	-
calmness	-0.291 (0.242)	-	-0.202 (0.169)	-	-0.338(0.120)*	-
instability of valence	0.396(0.191)*	0.511 (0.205)*	0.213 (0.159)	-	0.209 (0.118)	-
instability of energetic arousal	0.156 (0.147)	-	0.165 (0.129)	-	0.064 (0.099)	-
instability of calmness	0.229 (0.131)	-	0.214 (0.119)	-	0.089 (0.091)	-
**cognitive state variables (AA)**^d^						
rumination	0.275 (0.162)	-	0.185 (0.132)	-	0.283(0.093)**	-
rumination*time^e^	-	-	-	-	0.290(0.127)*	**-**
instability of daily-life rumination	0.138(0.065)*	-	0.093 (0.057)	-	0.099(0.042)*	0.065 (0.031)*

^a^ cox regression model

^b^ linear regression model

^c^ mixed models (hierarchical linear models)

^d^ AA Ambulatory Assessment variable

^e^ model includes main effects and interaction effect of predictor and time

^f^ all multiple models included subsample status as a covariate

*** *p* < .001

** *p* < .01

* *p* < .05

**Fig 2:**
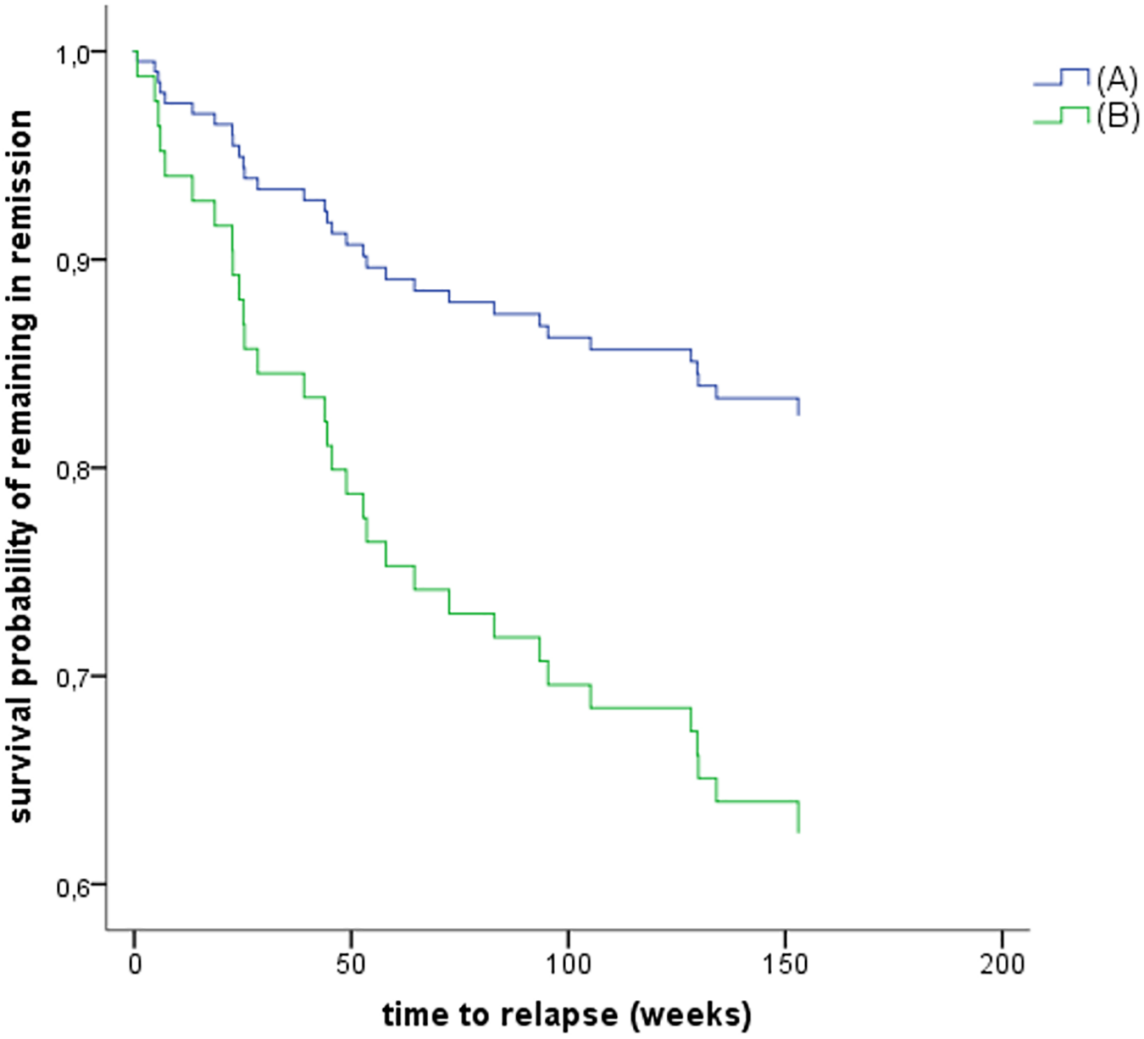
Estimated survival curve for remaining in remission in rMDD individuals with low and high instability of affective valence at baseline. (A) rMDD participants with low instability of affective valence (n = 28). (B) rMDD participants with high instability of affective valence (n = 28). Median split for illustrative purposes. Data from one participant were missing.

Fig 2. shows the corresponding survival curve (remaining in remission), estimated by the product-limit method of Kaplan and Meier, for rMDD individuals with low (A) and high instability (B) of affective valence at T1. In the multiple model, residual symptoms at baseline (B = .585, SE = .187, Wald = 9.7, *p* = .002), and higher instability of momentary valence (AA, B = .511, SE = .205, Wald = 6.1, *p* = .013) remained as independent significant predictors of time to relapse in the model.

### Predictors of chronicity

Higher levels of residual symptoms at baseline, more trait RNT, and lower levels of momentary energetic arousal (AA) were associated with a higher percentage of weeks spent with significant symptoms in the simple regression analyses (Table 2). In the multiple analysis, only higher levels of trait RNT (B = .032, SE = .009, t = 3.37, *p* = .001) remained in the final model (Table 2).

### Predictors of depressive symptom levels

Simple mixed model analyses revealed that higher levels of depression scores (T2, T3) were predicted by more severe residual symptoms, higher levels of trait RNT, higher momentary rumination (AA) and its interaction with time, higher instability of momentary rumination (AA), as well as by lower levels of momentary positive valence, energetic arousal and calmness (AA) during daily life. In the final multiple model, depression scores at baseline (B = .503, SE = .077, t = 6.52, *p* = < .001) and instability of daily-life rumination (AA) at baseline (B = .065, SE = .031, t = 2.11, *p* = .037) were retained as independent significant predictors.

## Discussion

The present longitudinal study investigated both trait and state predictors for the course of depression in a community sample of remitted depressed individuals with regard to risk of relapse, chronicity and symptom levels during an observation period of three years. We identified a rather unfavorable long-term depression course in our sample. During the 3-year follow-up, approximately half of the sample had suffered a relapse into a Major Depressive Episode, and nearly 30% had spent more than half of the follow-up period with significant depressive symptoms. Rates of relapses and chronicity are largely comparable to those found in previous research [3, 5, 58].

In the present study, a shorter time to relapse was predicted by higher residual symptom levels, higher instability of momentary mood (valence) and higher instability of rumination during daily life (AA) at baseline. In the multiple model, residual symptoms, and higher instability of momentary mood (AA) remained as significant independent predictors. Similar to our results, previous research has identified residual depressive symptomatology as a serious risk factor for relapse [2, 59, 60]. Given the high prevalence of residual symptoms during remission [61], even after successful treatment [39], this clearly deserves more attention in future relapse prevention efforts for depression. Regarding the predictive role of momentary affective state characteristics for the long-term course of illness, our results showed that a faster time to relapse was predicted by higher instability of momentary mood at baseline, thereby confirming our hypothesis. In previous studies, negative affect variability has been found to predict future negative affective symptoms in rMDD individuals [30] and treatment response in individuals with MDD [31]. Our longitudinal results confirm and expand these findings by showing that affective instability increases the vulnerability towards relapse [25, 29]. This finding has possible clinical implications. While current intervention programs aim to reduce the prolonged negative affect in acutely depressed individuals, fluctuations in everyday affect appear to be linked to a larger susceptibility for relapses or recurrences and could therefore specifically be considered in relapse prevention programs. Clearly, these considerations warrant more rigorous testing in future research.

Chronicity, i.e., the proportion of weeks spent with significant symptoms, was predicted by residual symptom levels, high levels of trait repetitive negative thinking (RNT) and low levels of momentary energetic arousal during daily life (AA) at baseline. In the multiple model, trait repetitive negative thinking was the most powerful and single remaining predictor. These results confirm our hypothesis suggesting that dysfunctional cognitive traits represent an important course moderating factor with regard to the development of a chronic course. Concordantly, high levels of habitual state orientation [60], dysfunctional attitudes [62] as well as ruminative thinking [10] have been found to be heightened in chronic depressed individuals or to predict a chronic symptom course.

Finally, depressive symptom levels at follow-up (T2, T3) were predicted by higher levels of residual symptoms and trait RNT, as well as by virtually all cognitive and affective state variables (AA). Residual symptom levels and instability of daily-life rumination (AA) remained as independent significant predictors in the multiple model. With respect to cognitive state variables (AA), we found that, contrary to our expectation, the instability of daily life rumination over the day (AA) was an even more important predictor for longer-term elevated levels of depressive symptoms than respective mean levels. A question is why higher levels of fluctuation in rumination could be maladaptive with this regard. Possibly, the instability of rumination was related to daily life stressors, which may have mediated the effect on depression symptom levels. However, this has not been investigated in the present study and therefore remains speculative. Our analyses do also not allow to decide whether possible emotional cascades, characterized by reciprocal cycles of rumination and negative affect, or pure cognitive fluctuations in the context of more stable elevated negative mood at baseline, predicted long-term elevated depressed symptoms (cf. [63, 64]). Further research is required on these aspects.

This study has several limitations. First, in order to include patients with a primary diagnosis of MDD, individuals with certain comorbid diagnoses were excluded, which may have led to a somewhat selective sample. Second, we combined samples from two substudies with a somewhat different baseline assessment design. To account for a possible influence of subsample status, we controlled for this variable and could not identify a possible confounding effect in any of the predictor analyses. Third, although dropout rates were satisfactorily low during the three-year period, we observed a selective dropout of rMDD individuals with lower education levels, thereby reducing the generalizability of our results. Fourth, although all rMDD participants had to be out of episode for at least two months before study inclusion, their residual symptom levels varied markedly. Fifth, we did not control for stressful daily-life events in the present analyses. Thus, it is difficult to conclude how much individual differences in the assessed affective and cognitive states were affected by environmental influences [25, 65]. Sixth, due to the investigated outcomes, the instability measures had to be aggregated, thereby eliminating the dynamic aspect at the within-subject level. However, MSSD still is a measure of instability, demonstrating differences in emotional fluctuations on the between-subject level [54, 57]. Finally, the multiple models showed that single predictors were correlated, and one could argue that the presentation of results from simple models is dispensable. However, we think that these results yield information about predictors that could be relevant for research questions in future studies, which may particularly apply to the daily life measures in hand.

To conclude, our results imply that illness-related characteristics and cognitive vulnerability traits, but also affective and cognitive state variables assessed during daily life, impact the longer-term course of depression. While trait repetitive negative thinking particularly predicted longer-term chronicity, the assessed state variables partly differently affected relapses and levels of depressive symptoms. Our findings imply that particularly those rMDD individuals showing high fluctuations in daily-life affect are susceptible to relapses, while individuals with a more frequent unstable pattern of rumination over the day are especially prone to suffer from persistently elevated symptoms. Importantly, these findings were not attributable to higher residual depression levels at baseline since this course-relevant clinical predictor was also retained in the respective final multiple models. Thereby, the assessed state variables provided independent contributions for the prediction of outcomes.

The present findings have several implications. First, our longitudinal results suggest that mood and rumination instability during daily life, indicating specific patterns of emotional dysregulation [25, 29], might reflect a kind of scarring process (cf.[20]) in patients with former episodes, which–per se or in combination with other risk factors–increases vulnerability towards relapse and maintenance of depressive symptoms. In fact, both variability parameters were significantly associated with the number of anamnestic MDE episodes in that the more depressive episodes an individual had experienced in the past, the higher were the current levels of mood and rumination instability. This finding is in line with a more dimensional view on scars, suggesting a gradual development of vulnerability factors with every MDD episode [20]. Connected herewith, it has been proposed that ambulatory assessment enables to assess basic regulatory processes such as stress reactivity, reward dependence, and affective instability, which may be relevant for a number of mental disorders [66]. As regards the latter, previous daily life research has investigated affective and cognitive instability features primarily in the context of other disorders such as bipolar disorder [67], borderline personality disorder [28, 29], and non-suicidal self-injury [63, 68]. The present study suggests that instability features may also play a role as course-relevant modifiers in major depression, thereby lending support for their role as transdiagnostic endophenotypes (cf.[66]). Future longitudinal research testing the role of these AA-phenotypes across mental disorders is clearly warranted. Finally, the study of the dynamic interplay of momentary cognitive and affective variables at the micro-level may help to even better understand determinants of the course of macro-level symptoms and diagnosis [18, 20].

## Supporting information

S1 File PONE-D-16-35985 personlevel-T1T2T3.sav. (SAV)

S2 File PONE-D-16-35985 personperiod-T2T3.sav. (SAV)

S1 Text Experimental procedure at baseline (T1). (DOCX)
