# Supplementary material for: Cognitive and affective trait and state factors influencing the long-term symptom course in remitted depressed patients
Source: PLoS One. 2017 Jun 2;12(6):e0178759. doi: 10.1371/journal.pone.0178759 (PMC5456349; doi:10.1371/journal.pone.0178759)
Supplement: S1 Text — (DOCX) [file pone.0178759.s003.docx]

**S1 Supporting information**

The present study sample of rMDD participants consisted of two consecutively recruited subsamples (subsample 1: Oct 2010 to Apr 2011, subsample 2: Nov 2011 to Nov 2012) from an overarching study. The two subsamples underwent a partly different ambulatory assessment (AA) procedure. The AA of subsample 1 was restricted to the assessment of naturally occurring mood and rumination over the day during two assessment days, whereas in subsample 2 an additional rumination versus mindful self-focus manipulation during four days of AA was conducted. At each of the ten assessments per day, participants rated momentary mood (valence, calmness, energetic arousal), and ruminative self-focus (focus on feelings/ problems) as spontaneous momentary baseline ratings. At the second, third, sixth, eighth and tenth assessment of each day, there was a subsequent 3 min induction of either ruminative (two induction days) or mindful attention (two induction days) via smartphone screen (randomized cross-over design) in subsample 2, in which the individuals had to focus their attention on 10 ruminative or mindful statements, followed by another rating of momentary mood and ruminative self-focus (post-induction ratings). Data from this particular induction part of this study have not been published so far and can therefore not be referred to in the paper. To identify a possible confounding effect of subsample status we performed three sets of analyses.

A): The analyses on predictive effects of the AA variables in the present paper did only include the pre-induction ratings (baseline-ratings), not the post-induction ratings from subsample 2. We conducted hierarchical linear models to check whether the induction day had a significant effect on the mood and rumination pre-induction (baseline) ratings. All models included time and induction day (0=rumination induction, 1=mindful self-focus induction) as fixed effects, and random intercepts on the person level. Here, we identified no significant effects of induction day on momentary valence (B= -.04, SE=.06, t=-.623, *p*=.534), calmness (B=.01, SE=.07, t=.161, *p*=.872), energetic arousal (B=-.03, SE=.07, t=-.398, p=.690) and rumination (B=-.11, SE=.09, t=-1.25, *p*=.211).

B): We further included the variable “subsample” (1=subsample 1, 2=subsample 2) as a covariate in all analyses. In fact, this variable showed no significant effect on any of the investigated outcomes (time to relapse: B= .50, SE=.40, Wald=1.554, *p*=.213; chronicity: B= .40, SE=.28, t=-1.469, *p*=.148; symptom levels: B= -.05, SE=.16, t=-.327, *p*=.744).

C): We conducted additional analyses separately for all outcomes to investigate possible significant interaction effects between subsample status and the corresponding significant predictor variables. Here, we did not identify any significant interaction between respective predictors and subsample status for the outcomes time to relapse (instability of valence x subsample: B= -.251, SE=.40, Wald=.421, *p*=.516, residual symptoms x subsample: B= .317, SE=.40, Wald=.698, *p*=.403), chronicity (RNT x subsample: B= -.005, SE=.02, t= -0.237, *p*=.813), and symptom levels (instability of rumination x subsample: B= -.024, SE=.09, t=- -0.271, *p*=.787, residual symptoms x subsample: B= -.004, SE=.17, t=- 0.024, *p*=.981).

Altogether, the results of these three sets of control analyses (A): nonsignificant effect of induction day on momentary baseline outcomes, B): nonsignificant effect of subsample status on any of the long-term outcomes after baseline (T1), C): nonsignificant interaction effect of subsample status and predictors on long-term outcomes) showed no indication of a confounding effect of subsample status on any of the presented results, thereby justifying the combination of both subsamples for the present long-term analyses.
